# Efficacy of Global Leadership Initiative on Malnutrition as potential cachexia screening tool for patients with solid cancer

**DOI:** 10.1186/s12937-022-00829-2

**Published:** 2022-12-07

**Authors:** Mengmeng Song, Qi Zhang, Tong Liu, Meng Tang, Xi Zhang, Guotian Ruan, Xiaowei Zhang, Kangping Zhang, Yizhong Ge, Ming Yang, Wei Li, Minghua Cong, Kunhua Wang, Chunhua Song, Hanping Shi

**Affiliations:** 1 Department of Gastrointestinal Surgery/Clinical Nutrition, Capital Medical University Affiliated Beijing Shijitan Hospital, No.10 Tieyi Road Haidian Dist, Beijing, 100038 China; 2 Key Laboratory of Cancer FSMP for State Market Regulation, Beijing, 100038 China; 3 Beijing International Science and Technology Cooperation Base for Cancer Metabolism and Nutrition, Beijing, 100038 China; 4 The Second Affiliated Hospital and Yuying Children’s Hospital of Wenzhou Medical University, Wenzhou, 325000 China; 5 Cancer Center of the First Hospital of Jilin University, Changchun, Jilin, 130021 China; 6 Department of Comprehensive Oncology, National Cancer Center/National Clinical Research Center for Cancer/Cancer Hospital, Chinese Academy of Medical Sciences and Peking Union Medical College, Beijing, China; 7 Yunnan University, Kunming, 650091 China; 8 Department of Epidemiology and Statistics, Henan Key Laboratory of Tumor Epidemiology College of Public Health, Zhengzhou University, Zhengzhou, 450001 Henan China

**Keywords:** Cancer, Cachexia, GLIM, PG-SGA, Screening tools

## Abstract

**Purpose:**

Cachexia has a very high prevalence in patients with cancer, and lacks effective screening tools yet. Global Leadership Initiative on Malnutrition (GLIM) is a novel malnutrition assessment tool, with increased important roles in malnutrition diagnosis for patients with cancer. However, whether GLIM can be used as an effective screening tool remains unknown.

**Methods:**

We performed a multicenter cohort study including 8,478 solid tumor patients from 40 clinical centers throughout China. Cachexia was diagnosed based on the 2011 international cancer cachexia consensus. The receiver operating characteristic curves (ROC) and decision curve analysis (DCA) were developed to determine the efficacy and clinical net benefit of GLIM and Patient-Generated Subjective Global Assessment (PG-SGA) in the detection of cancer cachexia, respectively.

**Results:**

According to the consensus guidelines, 1,441 (17.0%) cancer patients were diagnosed with cachexia among 8,478 patients in the present study. The sensitivity of one-step GLIM and two-step GLIM for detecting cachexia were 100 and 88.8%, respectively, while that of PG-SGA was 86.2%. The accuracies of one-step GLIM and two-step GLIM reached 67.4 and 91.3%, which were higher than that of PG-SGA (63.1%). The area under the curves (AUCs) of one-step GLIM (0.835) and two-step GLIM (0.910) were higher than PG-SGA (0.778) in patients with cancer. The DCA also revealed that two-step GLIM had better clinical effect than PG-SGA between 20-50% threshold probabilities.

**Conclusion:**

GLIM could be used as an effective tool in screening cancer cachexia, two-step GLIM criteria show more accurate while one-step GLIM criteria is more sensitive.

**Trial registration:**

ChiCTR1800020329.

**Supplementary Information:**

The online version contains supplementary material available at 10.1186/s12937-022-00829-2.

## Introduction

Cachexia, a multifactorial syndrome, is characterized by severe weight loss, sarcopenia, fatigue and compromised appetite [1], which frequently occurs in cancers such as pancreatic, gastrointestinal, head, neck and lung cancer [2]. Cachexia appears in up to 80% of cancer patients, causing at least 20% of cancer-associated deaths [3, 4]. Patients with upper gastrointestinal and pancreatic cancer suffered from the highest prevalence (reaching 80%) of cachexia [5], which may result in poor quality of life and survival of patients. However, globally recognized diagnosis criteria for cancer cachexia are still limited.

Patients with malignant tumors were afflicted with malnutrition with an estimated prevalence ranging from 40 to 80% [6]. In fact, cachexia is a special form of disease-related malnutrition which is difficult to reverse through nutrition support compared with common malnutrition [1]. Although there is no globally recognized screening tool for cancer cachexia yet [7], previous studies have tried to explore the availability of nutrition screening or assessment tools for cancer cachexia, including Malnutrition Universal Screening Tool (MUST), Nutritional Risk Screening 2002 (NRS-2002), Malnutrition Screening Tool (MST), Short Nutritional Assessment Questionnaire (SNAQ) [8] and Patient-Generated Subjective Global Assessment (PG-SGA) [9]. Originally, these malnutrition assessment tools were developed to estimate whether a patient has malnutrition or malnutrition risk. NRS-2002, the first step of Global Leadership Initiative on Malnutrition (GLIM) [10], is a nutrition risk screening tool recommended by the European Society for Parenteral and Enteral Nutrition (ESPEN) for adult patients [11]. The MUST was developed to evaluate protein–energy malnutrition and the risk of malnutrition for adults in community population based on three independent indicators. The MST, a simple, quick, and reliable instrument, was developed to detect the risk of malnutrition for patients at admission. The MUST, MST, SNAQ, and NRS-2002 was screening tools for the risk of malnutrition. The PG-SGA was the most authoritative tool for malnutrition diagnosis in patients with cancer [12]. GLIM, a new diagnostic framework for malnutrition in 2016 [13], builds an international consensus around the diagnostic criteria for malnutrition in adults [14]. As a novel guideline, the essential roles of GLIM in malnutrition diagnosis have been proven in many recent studies [14–19]. However, the reliability of GLIM in cancer cachexia assessment and its effectivity compared to other screening tools remain unclear.

It is important to screen and control the progress of cancer cachexia early. The purpose of the present study was to evaluate whether GLIM could be used as a favorable cachexia screening tool, and further to compare the effects of one-step GLIM, two-step GLIM and PG-SGA in the cancer cachexia screening and to explore the clinical significance of GLIM in early screen of cancer cachexia, so as to achieve early prevention and intervention of cachexia, and try to prevent or reduce the occurrence of cachexia.

## Material and methods

### Study population

Data from the Investigation on Nutrition Status and its Clinical Outcome of Common Cancers (INSCOC) project of China (Asia) were obtained from 40 clinical centers throughout China. The trial was registered at http://www.chictr.org.cn under the registration number ChiCTR1800020329. All participants were followed up via in-person or telephone questionnaires to collect requisite information by specialized staff. The specific inclusion criteria are as follows: (1) ≥ 18 years of age; (2) length of hospital stay > 48 h; and (3) diagnosis of one of the following 16 types of locally or metastatic malignant solid tumors: lung cancer, gastric cancer, liver cancer, colorectal cancer, breast cancer, esophageal cancer, cervical cancer, endometrial cancer, nasopharyngeal carcinoma, pancreatic cancer, ovarian cancer, prostate cancer, bladder cancer, brain tumors, biliary tract malignant tumors or gastrointestinal stromal tumors. The exclusion criteria are as follows: (1) organ transplantation; (2) current pregnancy; (3) diagnosis of HIV infection or AIDS; (4) admission to the ICU at the beginning of recruitment; and (5) more than two hospitalizations during the investigation period. Written informed consent was obtained from all participants. The study protocol conformed to the ethical guidelines of the 1975 Declaration of Helsinki and was approved by the medical ethics committee of first affiliated hospital of Sun yat-sen University (Medical Research Audit (2013) No. 82).

### Data collection

Within the first 48 h after hospital admission, written informed consent was signed by all patients or their legal representatives, and a comprehensive interview of all patients was performed by a dietitian or clinician to obtain recent preoperative nutritional information, including NRS-2002 score, PG-SGA score, and Karnofsky Performance Score (KPS). Laboratory indicators were obtained from routine blood test. Anthropometric measurements included height, body weight, mid-arm circumference (MAC), mid-arm muscle circumference (MAMC), calf circumference (CC, left calf circumference), hand grip strength (HGS) and triceps skinfold thickness (TSF). HGS was measured using a hand dynamometer (Jamar Hand Dynamometer, IL, USA). The handle was adjusted individually to the size of the patient’s hand. The percentage of weight loss was calculated by comparing present weight to the corresponding weight over time (six-month interval). BMI was calculated as body weight (kilograms) divided by the square of body height (meters). Considering the effect of weight on HGS, body weight-standardized HGS (HGS/W) was adopted in the study. Having malnutrition risk were defined as NRS-2002 score ≥ 3, and malnourished patients with cancer was defined as PG-SGA score ≥ 4, respectively.

### Diagnosis of malnutrition based on GLIM

The details of GLIM diagnosis criteria were shown in Table S1. The parameters for malnutrition diagnosis and severity grading based on GLIM have been described previously [20]. The one-step GLIM criteria (GLIM-step1) was defined as the malnutrition directly diagnosed by GLIM criteria without nutrition risk screening (performed by NRS-2002). Two-step GLIM criteria (GLIM-step2) was defined as the malnutrition diagnosed by GLIM criteria after nutrition risk screening (performed by NRS-2002) [13]. The evaluation of weight loss was performed for the malnutrition severity grading according to the previous GLIM criteria [20]. Referenced cutoff values of low BMI for malnutrition stage were defined according to a previous study of the Asian population [21, 22]. The quantity of muscle was evaluated by MAMC and CC, and HGS/W represented the muscle function. For each sex, the fifth percentile (p^5^) and 15th percentile (p^15^) of the MAMC, CC and HGS/W were calculated respectively. Values < p^15^ and < p^5^ were defined as positive for stage I and stage II malnutrition, respectively [22].

### Diagnosis of cancer cachexia

Combined with the international consensus framework [1], cancer cachexia was diagnosed by the following diagnostic criteria: (1). weight loss of > 5% over the past 6 months (in the absence of simple starvation); (2). a BMI of < 18.5 kg/m^2^ (based on the criteria of Asia) plus > 2% weight loss; or (3) appendicular skeletal muscle index consistent with sarcopenia (males < 7.0 kg/m^2^; females < 5.4 kg/m^2^) and any degree of weight loss > 2%. Cancer cachexia was diagnosed if the patients met one at least aforementioned criterion.

### Statistical analysis

Mean (standard deviation, SD) and t-test were used to describe and compare continuous variables. Categorical variables were described as numbers (percentages) and analyzed by Pearson Chi-square analysis. Receiver operating characteristic (ROC) curves and decision curves analysis (DCA) were generated to evaluate the efficiency and clinical net benefit of screening tools for detecting cachexia. The ROC curves of different subgroups (tumor types and TNM stage) were performed to evaluate the efficiency of three tools for screening cancer cachexia in different subgroups. Subgroup analysis (age and sex) were performed to detect the efficiency of GLIM and PG-SGA for screening cancer cachexia. In addition, the sensitivity, specificity and accuracy of GLIM and PG-SGA were calculated. All tests were two-sided and *P* < 0.05 was regarded as statistically significant. All statistical analyses were performed by software SPSS version 21 (IBM, Armonk, NY, USA) and R (Version 3.6.3), involving R packages “survminer”, “survival”, “rmda” and “pROC”.

## Results

### Baseline characteristics

**Fig. 1:**
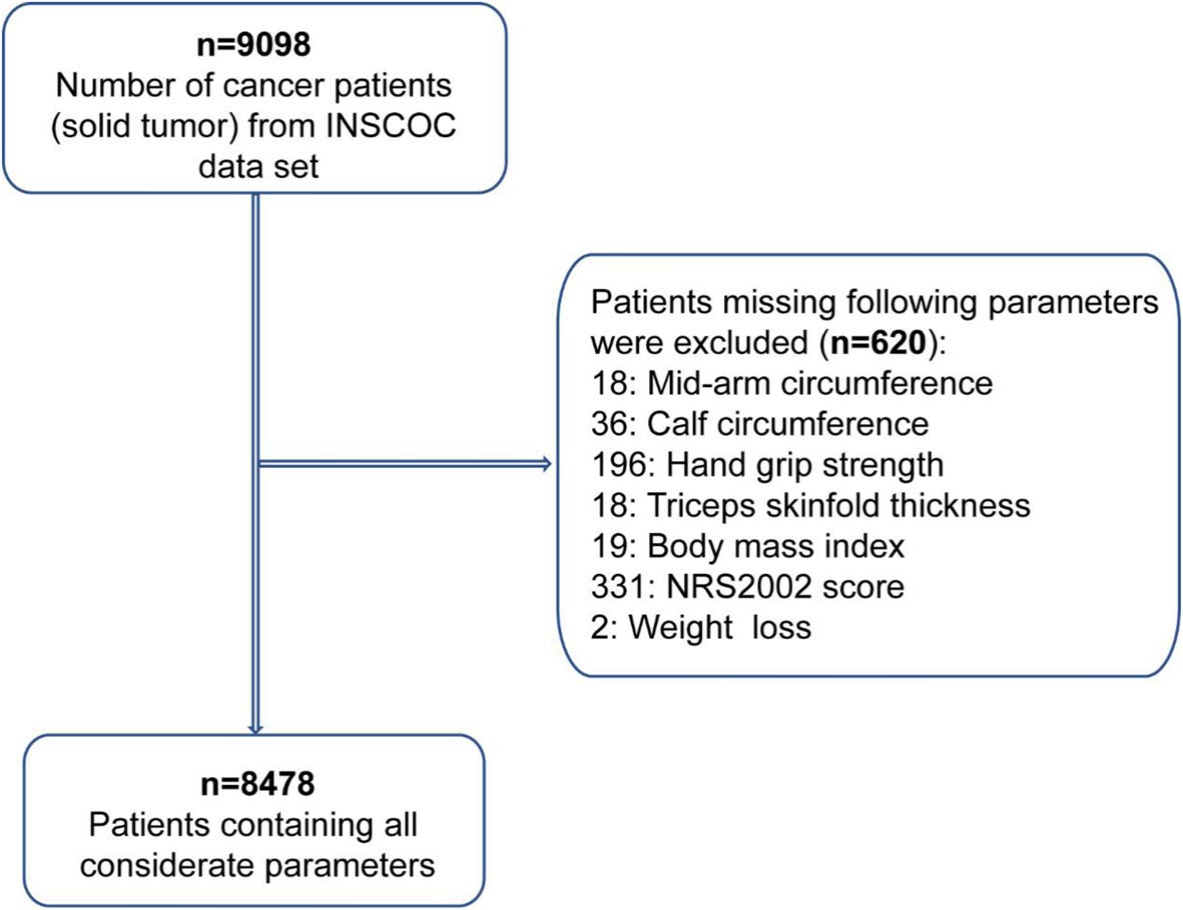
Flow chart. INSCOC, Investigation on Nutrition Status and its Clinical Outcome of Common Cancers

After exclusions of missing data, a total of 8,478 individuals were included in this study. The flow-chart of selection was shown in Fig. 1. Table1 gives the characteristics of the study population with or without cachexia. The mean age of the study population was 56.75 years old, with 4,338 (51.2%) males and 4,140 (48.8%) females. The patients with cancer cachexia were prone to have a significantly lower MAC, TSF, MAMC, CC and KPS than those in non-cachexia group (all *P* < 0.001). Patients with cancer cachexia tended to be younger than 65 years old, male, smoker, alcohol drinker, TNM stage III and IV, with lower albumin and higher NLR (all *P* < 0.001). In addition, compared with patients with cancer cachexia, non-cachexia group had more malnutrition diagnosed by GLIM-step1 and GLIM-step2, and had more anorexia (24.8% Vs. 10.4%) (*P* < 0.001).

**Table 1:** Baseline characteristics of the study population

	All cancer	Non-cachexia	Cachexia	
Characteristics	(*n* = 8478)	(*n* = 7037)	(*n* = 1441)	*P*
Age, y, mean (SD)	56.75 (12.05)	56.54 (11.83)	57.77 (13.01)	< 0.001
< 65	6218 (73.30)	5217 (74.10)	1001 (69.50)	< 0.001
≥ 65	2260 (26.70)	1820 (25.90)	440 (30.50)	
Sex, n (%)				
Male	4338 (51.20)	3465 (49.20)	873 (60.60)	< 0.001
Female	4140 (48.80)	3572 (50.80)	568 (39.40)	
BMI, kg/m^2^, mean (SD)	22.88 (3.47)	23.57 (3.06)	19.53 (3.39)	< 0.001
TNM, n (%)				
I	1261 (14.90)	1137 (16.20)	124 (8.60)	< 0.001
II	2052 (24.20)	1779 (25.30)	273 (18.90)	
III	2938 (34.70)	2388 (33.90)	550 (38.20)	
IV	2227 (26.30)	1733 (24.60)	494 (34.30)	
Smoke, yes, n (%)	3357 (39.60)	2675 (38.00)	682 (47.30)	< 0.001
Complication, yes, n (%)	2727 (32.2)	2266 (32.2)	461 (32.0)	0.901
Alcohol, yes, n (%)	1569 (18.50)	1222 (17.40)	347 (24.10)	< 0.001
Anorexia, yes, n (%)	1092 (12.90)	734 (10.40)	358 (24.80)	< 0.001
NRS-2002, ≥ 3, n (%)	2217 (26.20)	938 (13.30)	1279 (88.80)	< 0.001
PGSGA, n (%)				
0–3	4304 (50.80)	4105 (58.30)	199 (13.80)	< 0.001
4–8	2594 (30.60)	2093 (29.70)	501 (34.80)	
≥ 9	1580 (18.60)	839 (11.90)	741 (51.40)	
GLIM-step1, n (%)				
Well nourished	4271 (50.40)	4271 (60.70)	0 (0.00)	< 0.001
Moderate malnutrition	2800 (33.00)	1992 (28.30)	808 (56.10)	
Severe malnutrition	1407 (16.60)	774 (11.00)	633 (43.90)	
GLIM-step2, n (%)				
Well nourished	6624 (78.10)	6462 (91.80)	162 (11.20)	< 0.001
Moderate malnutrition	1122 (13.20)	425 (6.00)	697 (48.40)	
Severe malnutrition	732 (8.60)	150 (2.10)	582 (40.40)	
KPS, Mean (SD)	87.09 (12.79)	88.12 (11.69)	82.04 (16.28)	< 0.001
Albumin (g/L), Mean (SD)	39.53 (10.49)	39.89 (8.52)	37.78 (17.01)	< 0.001
≥ 35	6883 (81.20)	5924 (84.20)	959 (66.60)	< 0.001
< 35	1595 (18.80)	1113 (15.80)	482 (33.40)	
NLR, Mean (SD)	3.70 (7.15)	3.5 (6.64)	4.69 (9.19)	< 0.001
MAC (cm), Mean (SD)	26.58 (3.55)	27.08 (3.31)	24.16 (3.69)	< 0.001
TSF (mm), Mean (SD)	16.90 (8.01)	17.79 (7.89)	12.59 (7.19)	< 0.001
MAMC (cm), Mean (SD)	20.83 (3.43)	21.03 (3.42)	19.88 (3.27)	< 0.001
CC (cm), Mean (SD)	33.23 (3.91)	33.72 (3.68)	30.80 (4.07)	< 0.001

All of other cancer were solid neoplasms, including bladder cancer, prostate cancer, endometrial cancer, brain malignant tumor, gastric stromal tumor and biliary tract cancer

*SD* Standard deviation, *BMI* Body mass index, *NRS2002* the Nutritional Risk Screening 2002, *PG-SGA* Patient-Generated Subjective Global Assessment, *GLIM* the Global Leadership Initiative on Malnutrition, *GLIM-step1* One-step GLIM criteria, *GLIM-step2* Two-step GLIM criteria. One-step GLIM criteria and two-step GLIM criteria represented different GLIM criteria with or without nutrition risk screening by NRS-2002, respectively, *KPS* Karnofsky Score, *NLR* Neutrophil-to-lymphocyte ratio, *MAC* Mid-arm circumference, *TSF* Triceps skinfold thickness, *MAMC* Mid-arm muscle circumference, *CC* Calf circumference (left calf)

**Fig. 2:**
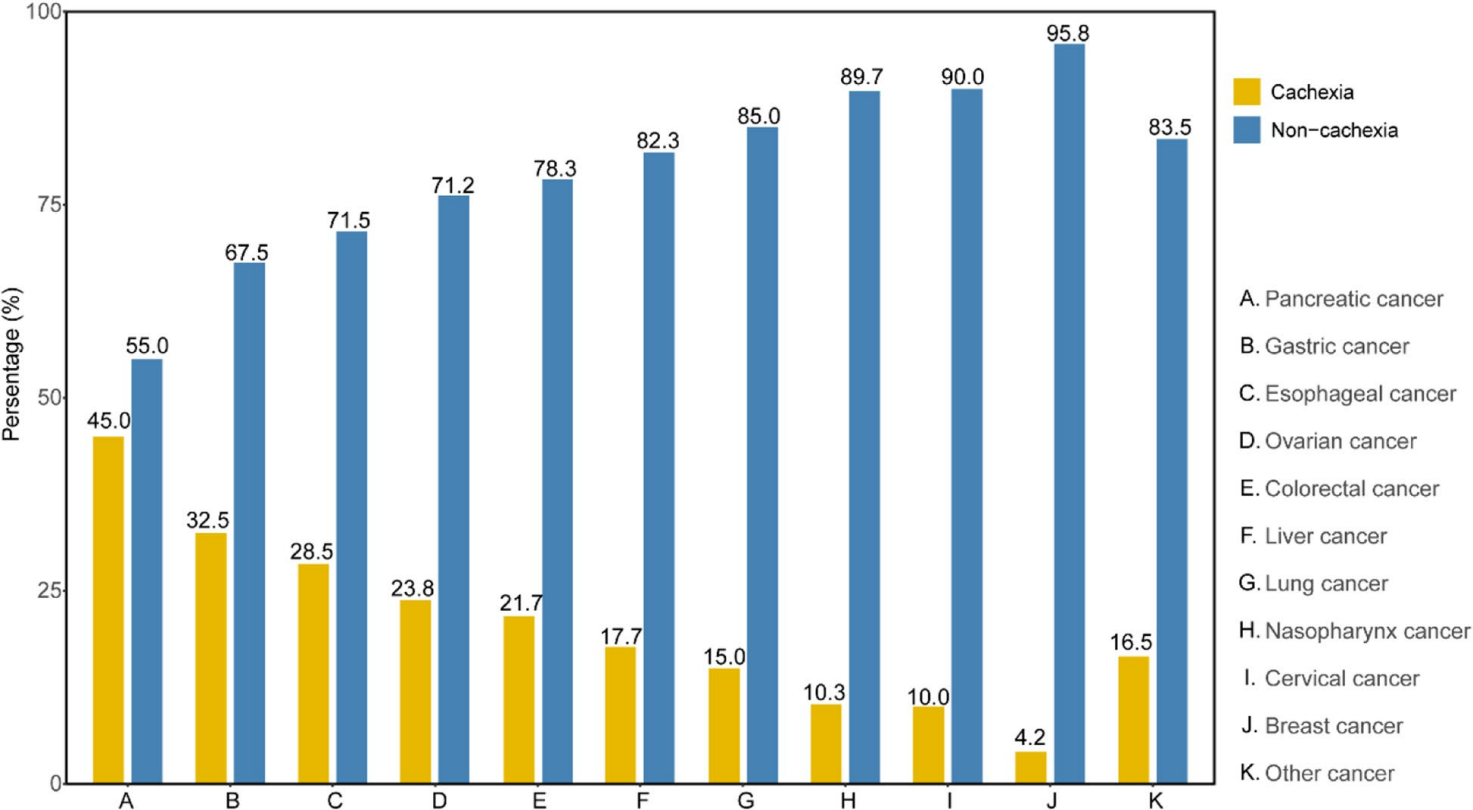
The cachexia prevalence in different types of cancer. Women cancer included breast cancer, ovarian cancer and cervical cancer; all of other cancers included bladder cancer, prostate cancer, endometrial cancer, brain malignant tumor, gastric stromal tumor and biliary tract cancer

Among all patients with cancer, pancreatic cancer was associated with highest cachexia prevalence accounting for 45.0%, followed by gastric cancer (32.5%), esophageal cancer (28.5%), ovarian cancer (23.8%) and colorectal cancer (21.7%), the details were shown in Fig. 2.

### The efficacy of GLIM in the screening of cancer cachexia

**Fig. 3:**
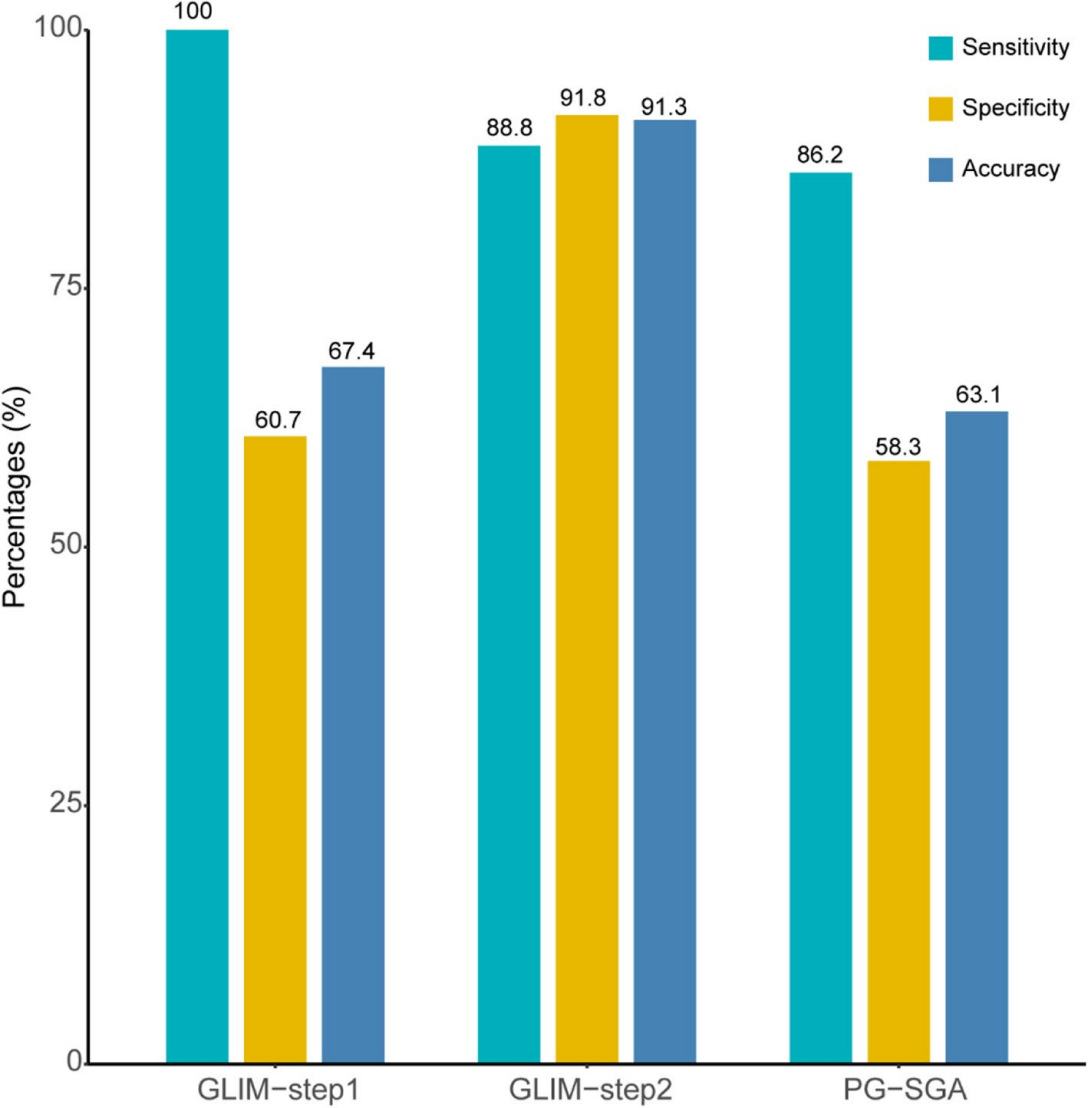
The sensitivity and specificity of GLIM and PG-SGA for detecting cancer cachexia. GLIM, Global Leadership Initiative on Malnutrition; PG-SGA, Patient-Generated Subjective Global Assessment. GLIM-step1: one-step GLIM criteria; GLIM-step2; two-step GLIM criteria. Two-step GLIM criteria and one-step GLIM criteria represented different GLIM criteria with or without nutrition risk screening by NRS-2002, respectively

**Fig. 4:**
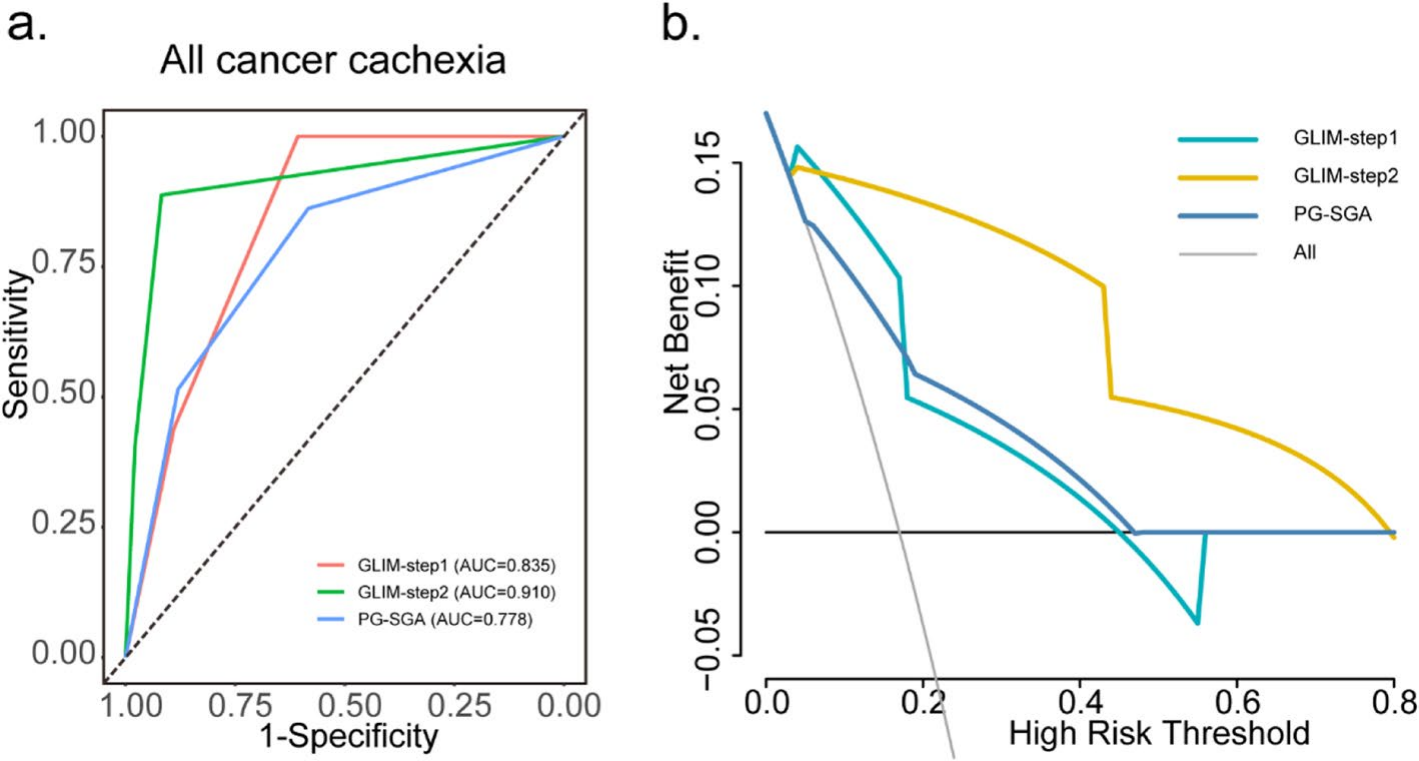
The ROCs and decision curve in predicting cachexia assessed by the PG-SGA and GLIM. **a** The ROCs of one-step, two-step GLIM criteria and PG-SGA for predicting all cancers patients with cachexia; **b** The decision curves for GLIM and PG-SGA to predict the correct diagnosis of cachexia in cancer patients. AUC, Area Under the Curve; GLIM, Global Leadership Initiative on Malnutrition; PG-SGA, Patient-Generated Subjective Global Assessment

The sensitivity of the GLIM-step1 and GLIM-step2 for detecting cancer cachexia among all cancer patients was 100% and 88.8% respectively, higher than PG-SGA (86.2%). Both the specificity and accuracy of GLIM-step1 and GLIM-step2 were higher than PG-SGA as shown in Table 2. Figure 3 showed the visualized sensitivities, specificities and accuracies of the GLIM and PG-SGA for detecting cancer cachexia. The AUC of GLIM-step2 was 0.910, which indicated better performance and a stronger capacity to identify patients with cachexia than GLIM-step1 (AUC = 0.835) and PG-SGA (AUC = 0.778) (Fig. 4a). The decision curves of the GLIM and PG-SGA showed that the clinical net benefit of GLIM-step2 was better than that of GLIM-step1 and PG-SGA between threshold probabilities of 20–50% (Fig. 4b). In the subgroup analysis, the AUC of GLIM-step2 was higher than that of GLIM-step1 and PGS-SGA in different cancer types (Supplementary Fig. 1), and TNM stages (Supplementary Fig. 2). There is the same trend in the subgroups of age (age < 65 and age ≥ 65) and BMI (BMI < 24 kg/m^2^ and BMI ≥ 24 kg/m^2^) (Supplementary Table 2).

**Table 2:** Sensitivity and specificity of the GLIM and PG-SGA for detecting cancer cachexia

	Cachexia	No Cachexia	Sensitivity	Specificity	Accuracy	
	(*n* = 1441)	(*n* = 7037)	(%)	(%)	(%)	AUC
GLIM-step1			100	60.7	67.4	0.835
Well nourished	0	4271	-	-		-
Malnutrition	1441	2766	-	-		-
GLIM-step2			88.8	91.8	91.3	0.910
Well nourished	162	6462	-	-		-
Malnutrition	1279	575	-	-		-
PG-SGA			86.2	58.3	63.1	0.778
Well nourished	199	4105	-	-		-
Malnutrition	1242	2932	-	-		-

*AUC* Area Under the ROC Curve, *GLIM* the Global Leadership Initiative on Malnutrition, *GLIM-step1* One-step GLIM criteria, *GLIM-step2* Two-step GLIM criteria. One-step GLIM criteria and two-step GLIM criteria represented different GLIM criteria with or without nutrition risk screening by NRS-2002, respectively; *PG-SGA* Patient-Generated Subjective Global Assessment, *PG-SGA* Well nourished (Score < 4), Malnutrition (Score ≥ 4)

## Discussion

In this multicenter population study, it was proved that GLIM can be used as a good tool for identifying cachexia in patients with cancer. One-step GLIM criteria had higher sensitivity than two-step GLIM criteria, but the specificity and final accuracy of two-step GLIM criteria was better than one-step GLIM criteria. ​Consequently, it is essential to perform a nutrition risk screening via NRS-2002 or other effective nutrition risk screening tools before the implementation of GLIM. Intriguingly, both one-step GLIM and two-step GLIM have favorable capacity than PG-SGA for detecting cachexia in patients with cancer. All cancer patients should be screened and evaluated for malnutrition risk after admission. Hence, the malnutrition diagnosis criteria, such as GLIM, can be used as a convenient tool for cachexia screening.

The international consensus framework for the definition and classification of cancer cachexia was developed [1] in 2011, declaring that weight loss, BMI and skeletal muscle depletion are the main factors for diagnosing cancer cachexia. Given that there were still no recognized diagnosis criteria of cancer cachexia yet, we used the international consensus framework as criteria of cachexia diagnosis in this research. The cutoff point of BMI in this study was based on the WHO recommended current cutoff points of Asia (18.5 kg/m^2^), instead of 20 kg/m^2^ in international consensus [23]. The skeletal muscle mass can be quantified with computed axial tomography (CT), magnetic resonance imaging (MRI), dual-energy x-ray absorptiometry, in vivo neutron activation analysis-whole body counting, ultrasound, bioimpedance analysis (BIA), and urinary metabolite markers [24, 25]. These methods were effective for diagnosing cancer cachexia. However, these methods can be costly and complex for cancer cachexia screening. We found that GLIM can be used as a simple screening tool of cancer cachexia, which can help early detection and timely intervention of cancer cachexia, prevent the progress of cachexia and improve the life quality of them.

Several tools for malnutrition assessment have been explored whether they can be used to screen for cachexia in patients with cancer. Among the assessment tools of MUST, NRS-2002, MST, SNAQ, MST was proved to have the greatest ability to detect cancer cachexia among patients with gastric cancer [8], but there is a lack of verification in other types of cancer patients. In addition, PG-SGA was also manifest good detective in cachexia screening for patients with cancer in a recent study [9], lacking comparison with other tools. Our present study assessed the nutritional status of 8,487 cancer patients from multi-centers throughout China by the GLIM and PG-SGA. We found that the GLIM can be a potential screening tool for cancer cachexia, compensating for the insufficiency of cancer types in a previous study. In addition, compared with PG-SGA and other malnutrition assessment tools, the GLIM has fewer items and is easier to perform.

Consistent with previous research [2], this study revealed that pancreatic cancer has a higher incidence of cachexia than other cancers, with gastroesophageal cancer ranked as the second. Interestingly, the prevalence of cachexia in male patients was higher than that in female patients in this study. This is presumably because the incidences of pancreatic cancer, stomach cancer, colorectal cancer and esophageal cancer were higher in males than in females [26, 27], and the largest number of cachexia diagnoses occurred in these several cancer types [2]. The prevalence of cachexia increased with the progression of TNM stage I-IV in this study. A previous study reported that cachexia can also occur in curable cancers and may be reversed by effective treatment [28]. Therefore, early and accurate screening of cancer cachexia is important for effective nutritional intervention and anticancer treatment response. The prevalence of cachexia in all solid cancer patients in our study was 17.0%, lower than the incidence of cancer cachexia in previous study [29], probably because many types of cancer patients were enrolled in this study, including breast cancer and nasopharynx cancer, which have a relatively low prevalence of cachexia. The process of GLIM-diagnosed malnutrition is more stringent than the diagnosis with PG-SGA, thus the number of cancer patients with PG-SGA-diagnosed malnutrition was greater than that of patients with GLIM-diagnosed malnutrition. In addition, compared with the sensitivity and specificity, the clinical net benefit is more essential for nutritional intervention for cancer cachexia. The DCAs revealed that the two-step GLIM had better clinical net benefit than one-step GLIM and PG-SGA.

There are several limitations in this study. Firstly, studying population barring hematologic tumor may generate selection bias, leading to the conclusion of our study was not suitable for hematologic tumor. Secondly, only two nutritional screening tools were involved in the comparison. Thus, GLIM needs to be verified and compared with more screening tools in future research.

## Conclusion

Our results demonstrated that GLIM is a potentially simple cachexia screening tool in patients with cancer. This finding may facilitate early detection and effective management of cancer cachexia in clinical practice. Further study in a larger population from different areas should be performed to validate the performance of GLIM in the cancer cachexia screening.

## Supplementary Information

**Additional file 1. Supplementary Figure.** The ROC curves in predicting cachexia in different cancers assessed by the PG-SGA and GLIM. (a-i). The ROCs of GLIM, PG-SGA for pancreatic cancer, gastric cancer, esophageal cancer, and colorectal cancer, lung cancer, liver cancer, nasopharynx cancer, women cancer and other cancer, respectively. Women cancer included breast cancer, ovarian cancer and cervical cancer; all of other cancers included bladder cancer, prostate cancer, endometrial cancer, brain malignant tumor, gastric stromal tumor and biliary tract cancer. **Supplementary Figure.** The ROCs of GLIM, PG-SGA for detecting cachexia in cancer patients with different TNM stages. (a-d). The ROCs of one-step GLIM, two-step GLIM and PG-SGA for detecting cachexia in patients with TNM stage I, stage II, stage III and stage IV, respectively.

**Additional file 2. Supplementary Table.** Phenotypic and etiologic criteria the diagnosis of malnutrition of GLIM. **Supplementary Table.** Subgroup analysis of the GLIM and PG-SGA for detecting cancer cachexia.

## Data Availability

Not applicable.
