# Supplementary figures and images for: Efficacy of Global Leadership Initiative on Malnutrition as potential cachexia screening tool for patients with solid cancer
Source: Nutr J. 2022 Dec 7;21:73. doi: 10.1186/s12937-022-00829-2 (PMC9727850; doi:10.1186/s12937-022-00829-2)

**Figure S1.**

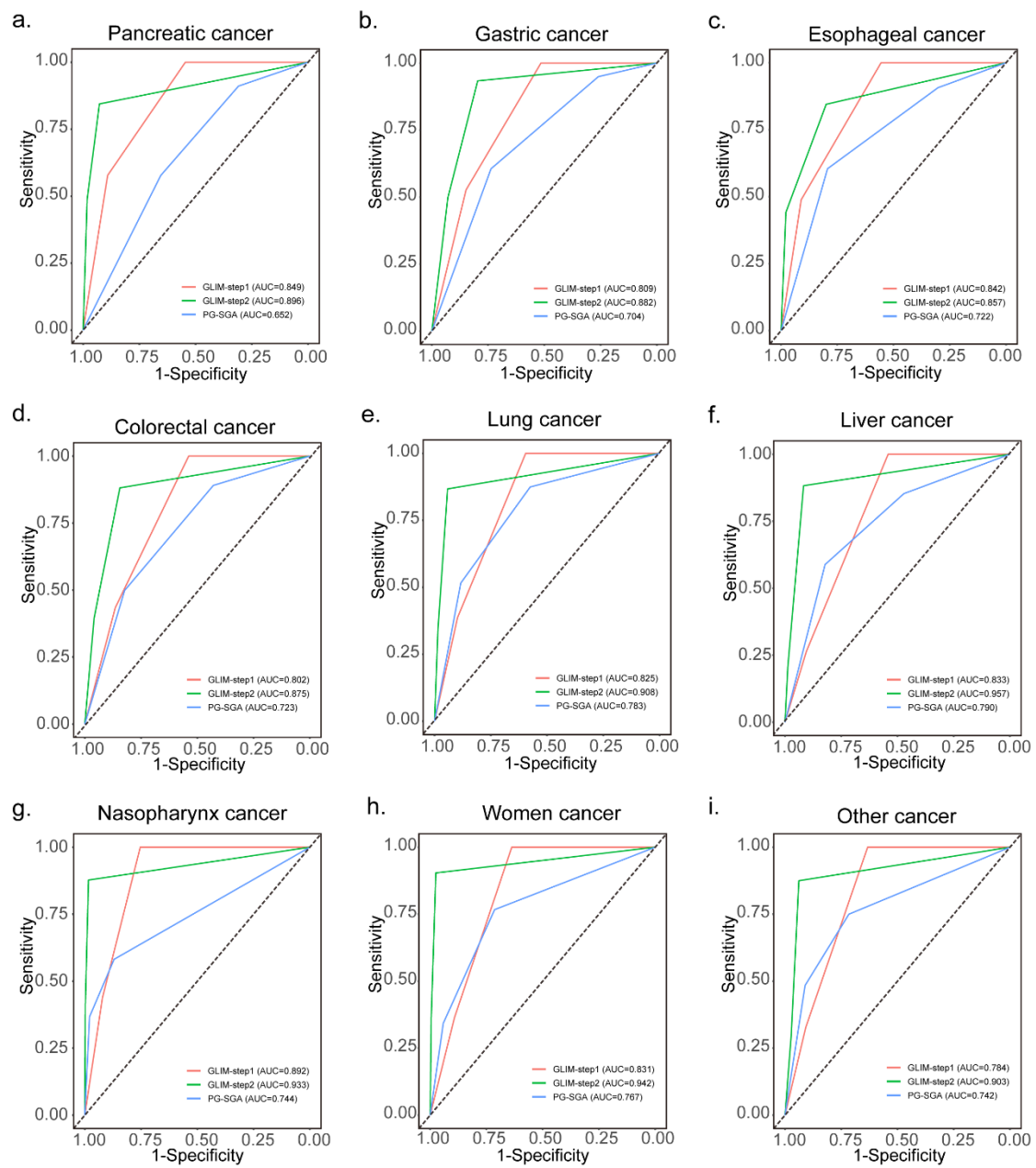

**Figure S2.**

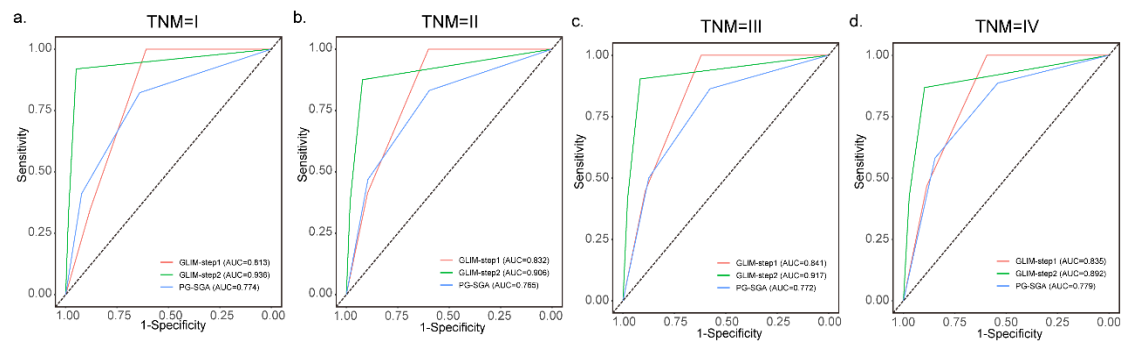

Supplement: Supplementary file 1 — Additional file 1. Supplementary Figure. The ROC curves in predicting cachexia in different cancers assessed by the PG-SGA and GLIM. (a-i). The ROCs of GLIM, PG-SGA for pancreatic cancer, gastric cancer, esophageal cancer, and colorectal cancer, lung cancer, liver cancer, nasopharynx cancer, women cancer and other cancer, respectively. Women cancer included breast cancer, ovarian cancer and cervical cancer; all of other cancers included bladder cancer, prostate cancer, endometrial cancer, brain malignant tumor, gastric stromal tumor and biliary tract cancer. Supplementary Figure. The ROCs of GLIM, PG-SGA for detecting cachexia in cancer patients with different TNM stages. (a-d). The ROCs of one-step GLIM, two-step GLIM and PG-SGA for detecting cachexia in patients with TNM stage I, stage II, stage III and stage IV, respectively. [file 12937_2022_829_MOESM1_ESM.pdf]
