# Supplementary material for: Efficacy of Global Leadership Initiative on Malnutrition as potential cachexia screening tool for patients with solid cancer
Source: Nutr J. 2022 Dec 7;21:73. doi: 10.1186/s12937-022-00829-2 (PMC9727850; doi:10.1186/s12937-022-00829-2)
Supplement: Supplementary file 2 — Additional file 2. Supplementary Table. Phenotypic and etiologic criteria the diagnosis of malnutrition of GLIM. Supplementary Table. Subgroup analysis of the GLIM and PG-SGA for detecting cancer cachexia. [file 12937_2022_829_MOESM2_ESM.pdf]

Supplementary Table 1. Phenotypic and etiologic criteria the diagnosis of malnutrition of GLIM.

Phenotypic Criteria			Etiologic Criteria
Weight loss（%）	Low BMI	Reduced Muscle mass	
>5% within past 6 months	Asia:<br><18.5 if<70y<br><20 if >70y	MAMC < p^{15}<br>HGS/W < p^{15}<br>CC < p^{15}	Disease burden

GLIM, the Global Leadership Initiative on Malnutrition; BMI, body mass index; MAMC, mid-arm muscle circumference; HGS/W, the body weight-standardized hand grip strength; CC, calf circumference (left calf).

Supplementary Table 2. Subgroup analysis of the GLIM and PG-SGA for detecting cancer cachexia

	AUC			
	Age<65	Age ≥65	BMI<24	BMI ≥24
GLIM-step1	0.8508	0.7992	0.8289	0.8156
GLIM-step2	0.9091	0.9119	0.9095	0.8656
PG-SGA	0.7858	0.7587	0.7469	0.8476

AUC: Area Under the ROC Curve; BMI, body mass index; GLIM, the Global Leadership Initiative on Malnutrition; GLIM-step1: one-step GLIM criteria; GLIM-step2; two-step GLIM criteria; One-step GLIM criteria and two-step GLIM criteria represented different GLIM criteria with or without nutrition risk screening by NRS-2002, respectively; PG-SGA, Patient-Generated Subjective Global Assessment.
